# Covariation in the recognition of own-race and other-race faces argues against the role of group bias in the other race effect

**DOI:** 10.1038/s41598-022-17330-9

**Published:** 2022-07-29

**Authors:** Ao Wang, Craig Laming, Timothy J. Andrews

**Affiliations:** Department of Psychology, University of York, York, YO10 5DD UK

**Keywords:** Neuroscience, Psychology

## Abstract

A dominant theory of the other race effect (ORE) is that group-bias causes us to process own-race and other-race faces using different cognitive processes. To test this theory, we measured individual differences across two face recognition tasks. Our predictions were that the magnitude and pattern of performance on own-race faces would not predict performance on other-race faces and that participants would take more time with own-race faces. In a face matching task, we found that participants were more accurate with own-race faces compared to other-race faces. However, performance on own-race faces was highly correlated with performance on other-race faces. In a face sorting task, participants made fewer piles and fewer errors (i.e. higher accuracy) with own-race faces compared to other-race faces. However, we again found that performance on own-race faces was highly correlated with performance on other-race faces. The covariation in performance between own-race and other-race faces suggests that they engage similar perceptual processes. Finally, we found that participants did not spend more time on tasks involving own-race faces suggesting that different levels of motivation do not explain the ORE. Together, these findings argue against the idea that group bias leads to different perceptual processing of own-race and other-race faces.

## Introduction

The other-race effect is a well-established phenomenon in face perception in which own-race faces are perceived more accurately than other-race faces^1^. The ORE has since been demonstrated using a wide range of protocols and cultural settings^2^. The majority of studies reporting the ORE have investigated face memory^3–5^. However, the ORE is also evident in perceptual tasks, such as matching^6–8^, sorting^9^ and discrimination^10,11^, showing that it must also involve the encoding of faces.

Despite its robustness, the ORE has defied a simple explanation. One theory, founded in social identity theory^12^, suggests that own-race and other-race faces are processed in fundamentally different ways. Own-race faces due to their in-group status are processed at an individual level, whereas other-race faces due to their out-group status are processed at a categorical level^13–15^. Thus, the processing of own-race and other-race faces is different depending on the outcome of the preceding categorization^16^. Support for this theory comes from studies that show other-race faces are more efficiently categorized than own-race faces, whereas own-race faces are more efficiently individuated^17^. Other support for a group bias account of the ORE comes from studies that show that group differences that are not based on race can also lead to differences in face recognition similar to the ORE^15,18–21^.

An alternative theory of the ORE proposes that the same-race advantage results from greater experience with own race faces^22–26^. Because of the higher exposure to own-race faces, the visual system becomes more ‘tuned’ to differentiate between individual own race compared to individual other-race faces^27,28^. The role of experience is shown in developmental studies which show an increase in the ORE with experience^29,30^ and by the fact that the ORE can be reversed or reduced if one is exposed to another racial group during development^31,32^. Although this theory predicts better performance for own-race compared to other-race faces, it also predicts that the same processes will be used for the recognition of own-race and other-race faces.

**Figure 1:**
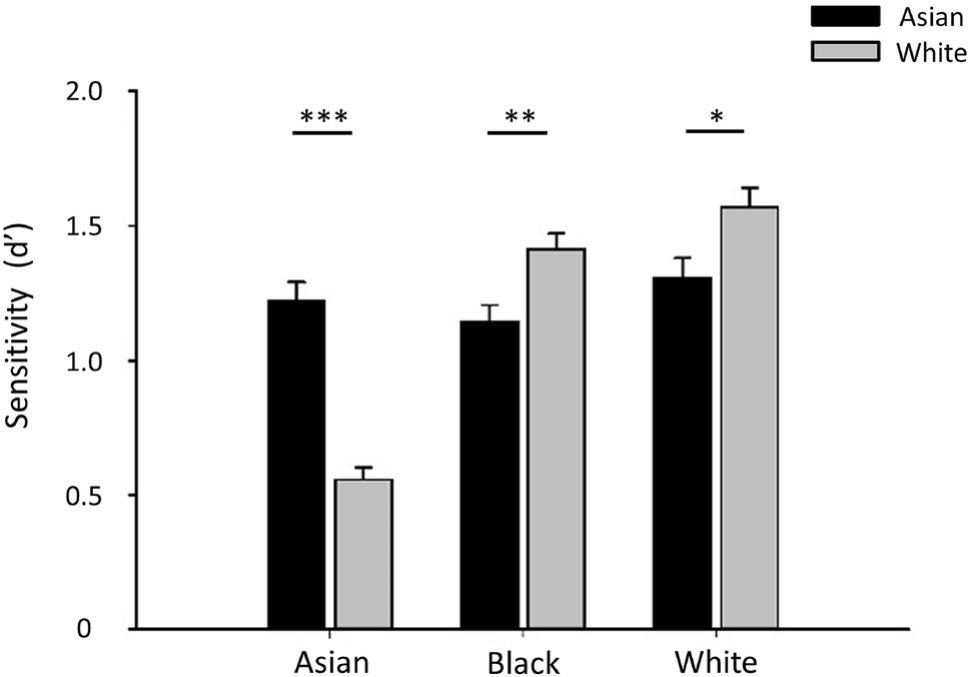
Performance (d’) on the matching task with Asian and White participants viewing Asian, Black and White faces. The data show a clear ORE with higher performance on own-race compared to other-race faces. Error bars show + 1 SEM. ***p < 0.001, **p < 0.01, * p < 0.05.

**Figure 2:**
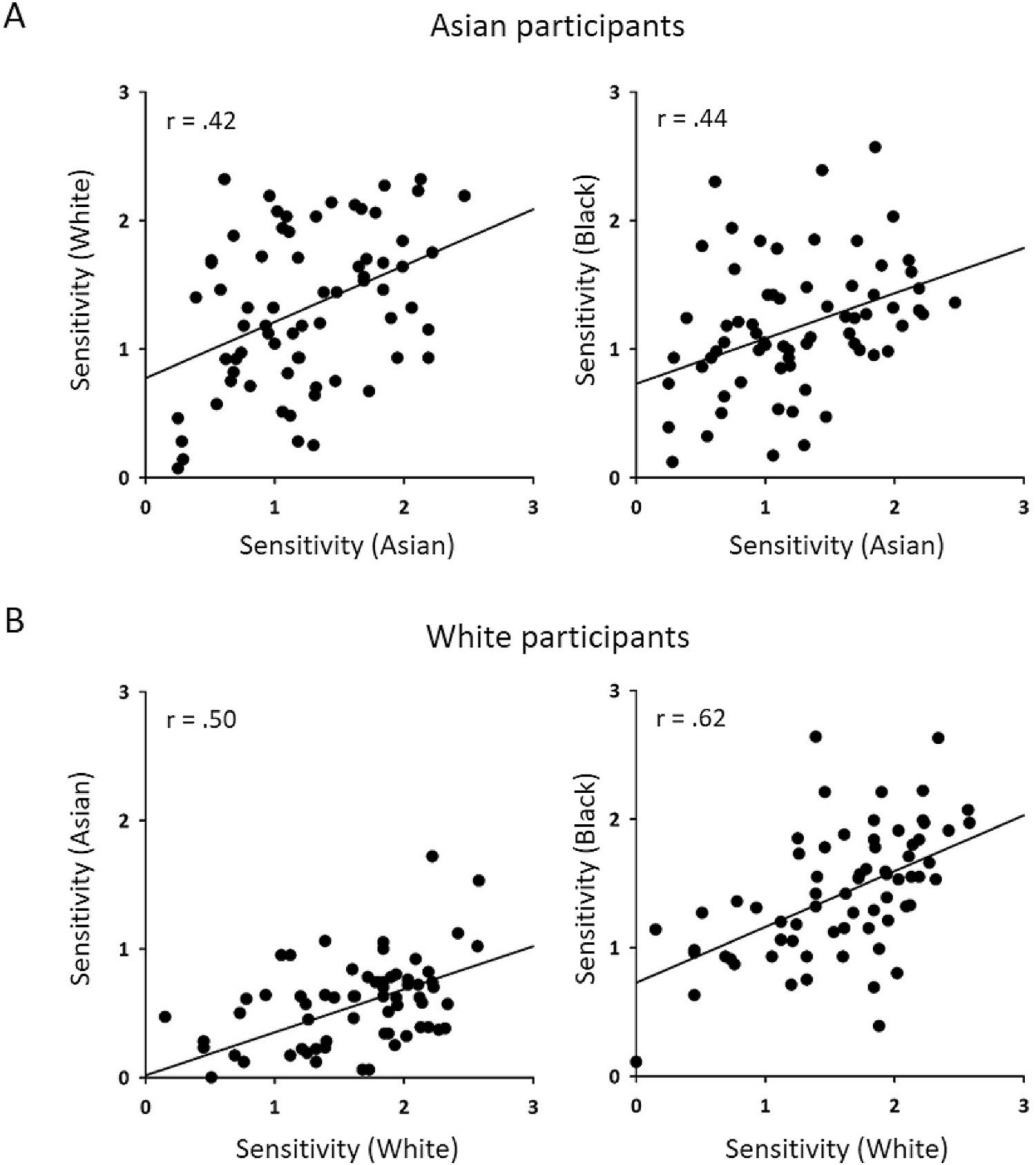
Correlation between d’ values between different face matching tasks in Asian and White participants. Significant positive correlations were found for each matching task for both own-race and other-race faces suggesting that performance on own-race faces predicted performance on other-race faces.

The aim of this study was to differentiate between these different explanations of the ORE. To do this, we used an individual differences approach to determine whether performance with own-race faces predicts performance on other-race faces. Previous studies have reported covariation in performance on own-race and other-race faces^7,8,33–36^. However, these findings could be explained by variation in motivation or general perceptual ability. To address this issue, we recruited a large population of Asian and White participants and tested them on two different perceptual tasks of face recognition: matching (Fig. 1) and sorting (Fig. 2). These tasks require participants to determine (i) whether faces are from the same person (putting faces together) and (ii) whether they are from different people (telling faces apart). For example, accuracy on same-identity trials in the matching task measures the ability to ‘put faces together’, whereas performance on the different-identity task measures the ability to ‘tell faces apart’. Similarly, the numbers of piles in the sorting task reflects variation in the ability to ‘put faces together’, whereas errors in which images of different identities are included in the same pile reflects the ability to ‘tell faces apart’. Our aim was to show whether covariation was evident across these potentially independent subprocesses of face recognition. All tasks were self-paced, but we measured the time taken to complete each task.

A prediction from social cognitive accounts of the ORE is that overall performance on own-race faces will not be highly predictive of performance on other-race faces, as they engage different cognitive processes. Similarly, the pattern of performance across items on own-race faces will not predict the pattern for other-race faces. Finally, participants should spend more time on tasks with own-race (in-group) faces compared to other-race (out-group) faces. On the other hand, the prediction from perceptual experience accounts of the ORE is that the same perceptual processes are involved. Therefore, the overall performance and the pattern of response to own-race faces would predict overall performance and the pattern of response to other-race faces.

## Results

### Matching task

Figure 1 shows the average performance of Asian and White participants in the matching tasks. There was a significant interaction between stimulus race and participant race (F(2, 279) = 65.539, *p* < 0.001, Partial Eta Squared = 0.32). This was due to higher performance on own-race faces in both Asian and White participants. For the Asian face matching task, there was a significantly higher d’ in Asian participants compared to White participants (t(69) = 7.81, *p* < 0.001, d = 1.36). However, on the White face matching task, there was a significantly higher d’ for White participants compared to Asian participants (t(69) = 3.15, *p* < 0.01, d = 0.52). For Black faces, White participants had a significantly higher d’ compared to Asian participants (t(69) = 2.81, *p* < 0.01, d = 0.50). The higher recognition of Asian faces in Asian participants and White faces in White participants provides clear evidence of an ORE. We also found that there was a negative correlation in Asian participants between the difference in d’ for Asian faces compared to White faces with the time spent in the UK (r = − 0.297, p = 0.012). That is, the ORE was lower in participants who had spent more time in the UK.

Next, we used an individual differences approach to determine whether performance on own-race faces predicted performance on other-race faces. We found performance on own-race faces was positively correlated with other-race faces (Fig. 2). For Asian participants, sensitivity for Asian face matching was positively correlated with accuracy on Caucasian (*r*_*s*_ = 0.421, *p* < 0.001) and Black (*r*_*s*_ = 0.440, *p* < 0.001) face matching. For Caucasian participants, sensitivity for White face matching was positively correlated with Asian (*r*_*s*_ = 0.499, *p* < 0.001) and Black (*r*_*s*_ = 0.617, *p* < 0.001) face matching. This suggests that performance on own-race faces predicts performance on other-race faces.

The d’ analysis combines performance on same and different identity trials. In the next analysis, we asked if the ORE was evident for performance on both same identity trials (‘putting faces together’) and different identity trials (‘telling faces apart’) independently. To determine if there was any bias in the pattern of response on same and different trials, we measured the proportion of Same and Different answers that our participants regardless of accuracy. Asian and White participants gave a similar proportion of same responses (Asian faces: Asian = 46.7%, White = 47.4%; Black faces: Asian = 46.1%, White = 45.0%; White faces: Asian = 46.5%; White = 47.0%). Next, an ANOVA with Face Race (Asian, Black, White) and Participant Race (Asian, White) as factors was run separately for accuracy on the same identity and different identity tasks. There was a significant interaction of Face Race * Participant Race for both same identity (F(2, 276) = 33.81, *p* < 0.001, Partial Eta Squared = 0.20) and different identity (F(2, 276) = 41.96, *p* < . 001, Partial Eta Squared = 0.23) faces. For Asian faces, the accuracy of Asian participants was greater than for White participants with both same identity ((Asian mean: 0.68, White mean = 0.58; t(138) = 5.02, *p* < 0.001, d = 0.85) and different identity (Asian mean = 0.75, White mean = 0.63; t(138) = 4.88, *p* < 0.001, d = 0.83) face trials. For White faces, accuracy was higher for White compared to Asian participants on both same identity (White mean = 0.74, Asian mean = 0.69; t(138) = 1.89, p = 0.061, d = 0.32) and different identity (White mean = 0.79: Asian mean = 0.75; t(138) = 1.79, p = 0.075, d = 0.30) face trials, but this failed to reach significance. For Black faces, although there was no significant difference in accuracy for Asian and White participants for the same identity trials (Asian mean = 0.66, White mean = 0.69; t(138) = 1.50, p = 0.14, d = 0.25), there was a significant difference on different identity trials (Asian mean = 0.76, White mean = 0.79; t(138) = 2.23, *p* < 0.05, d = 0.38). Together, these results show that performance on both same identity and different identity trials is biased toward own-race faces. However, similar to the d’ analysis (see Fig. 4), performance on own-race faces predicted performance on other-race faces for both same-identity and different identity trials (Table 1).

**Table 1:** Correlation between accuracy on own-race and other-race faces on same identity and different identity trials. The results show that performance on own-race faces strongly predicted performance on other-race faces.

		Same identity		Different identity	
		*r*_*s*_	*p*	*r*_*s*_	*p*
Asian participants	Asian/black	0.514	0.0001	0.566	0.0001
	Asian/white	0.515	0.0001	0.571	0.0001
	Black/white	0.636	0.0001	0.554	0.0001
White participants	Asian/black	0.665	0.0001	0.651	0.0001
	Asian/white	0.689	0.0001	0.641	0.0001
	Black/white	0.697	0.0001	0.663	0.0001

We then asked whether ability on same-identity (‘putting faces together’) is correlated with ability on different-identity trials (‘telling faces apart’). If performance on these measures are related, we would expect a significant positive correlation. For Asian participants, there was no significant correlation between same-identity and different-identity trials for Asian faces (*r*_*s*_ = − 0.023, p = 0.852). The correlation between performance on same and different trials for White faces was marginal (*r*_*s*_ = − 0.207, p = 0.086), but there was a significant negative correlation for Black faces (*r*_*s*_ = − 0.329, *p* < 0.01). For White participants, there was no significant correlation between same-identity and different-identity trials for White faces (*r*_*s*_ = − 0.095, p = 0.432). However, there were significant negative correlations in performance for same and different identity trials with Asian faces (*r*_*s*_ = − 0.445, *p* < 0.001) and Black faces (*r*_*s*_ = − 0.291, *p* < 0.05). Together, these findings that there is no reliable covariation between performance on same-identity and different-identity trials in the matching task.

**Figure 3:**
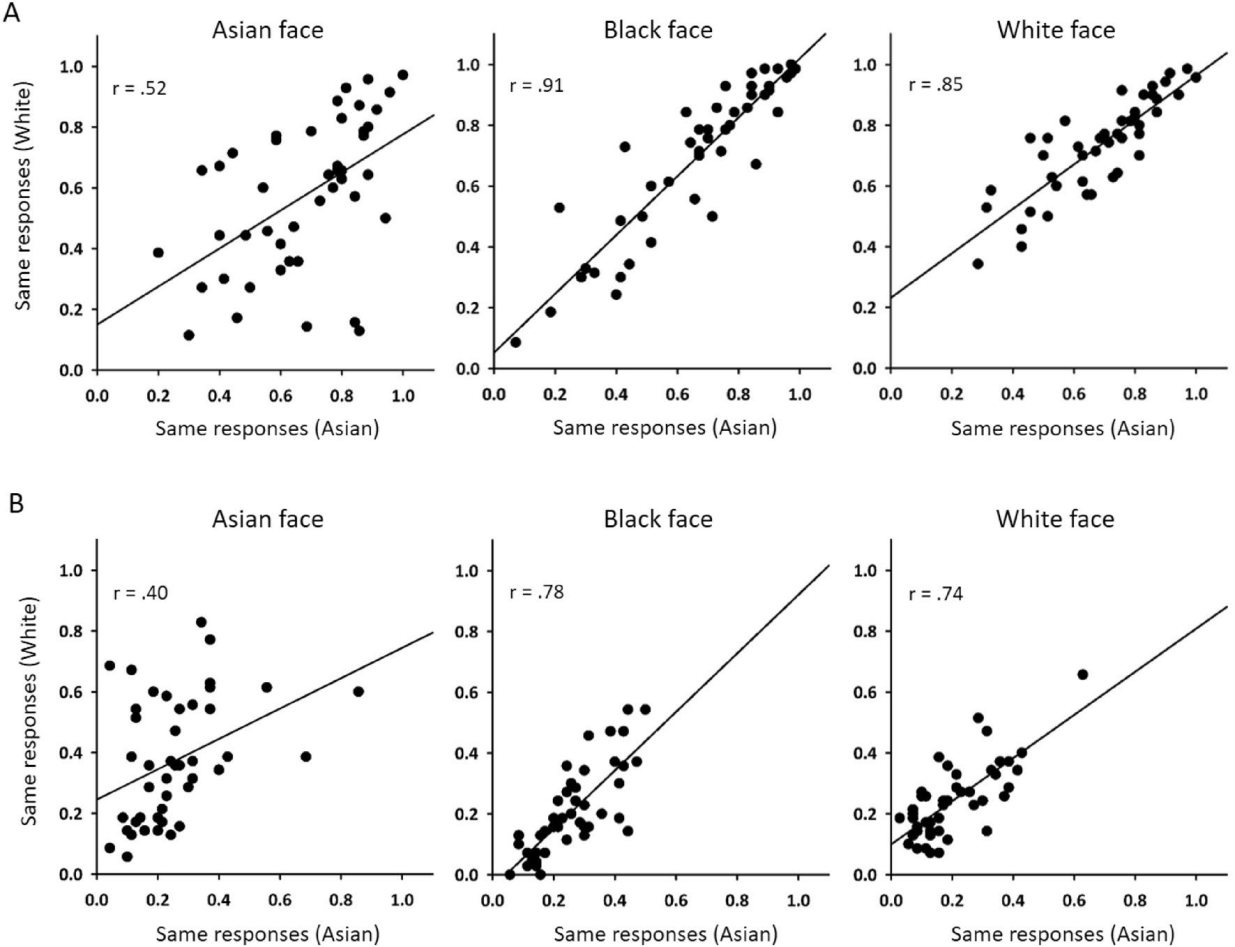
A comparison of an item-analysis across the two participant groups on (**A**) same identity and (**B**) different identity trials. Significant positive correlations show that Asian and White participants from different ethnicity made qualitatively similar responses on all matching tasks.

To determine whether there were differences in the way that individual trials were perceived by participants from different races, we performed an item-level analysis. We calculated the proportion of correct responses for each trial across Asian or White participants. This gave a vector of 45 values for the same-identity trials and a vector of 45 values for the different-identity trials for each task in each participant group. We then correlated these vectors for Asian and White participants (Fig. 3). For the same-identity trials, Asian and White participants had positive correlations across all tasks (Asian: *r*_*s*_ = 0.515, *p* < 0.001; Black: *r*_*s*_ = 0.909, *p* < 0.001; White: *r*_*s*_ = 0.844, *p* < 0.001). For the different-identity trials, Asian and White participants also had positive correlations across all tasks (Asian: *r*_*s*_ = 0.384, *p* < 0.01; Black: *r*_*s*_ = 0.776, *p* < 0.001; White: *r*_*s*_ = 0.735, *p* < 0.001). This shows that the pattern of response across trials or items is similar in participants from different races.

**Figure 4:**
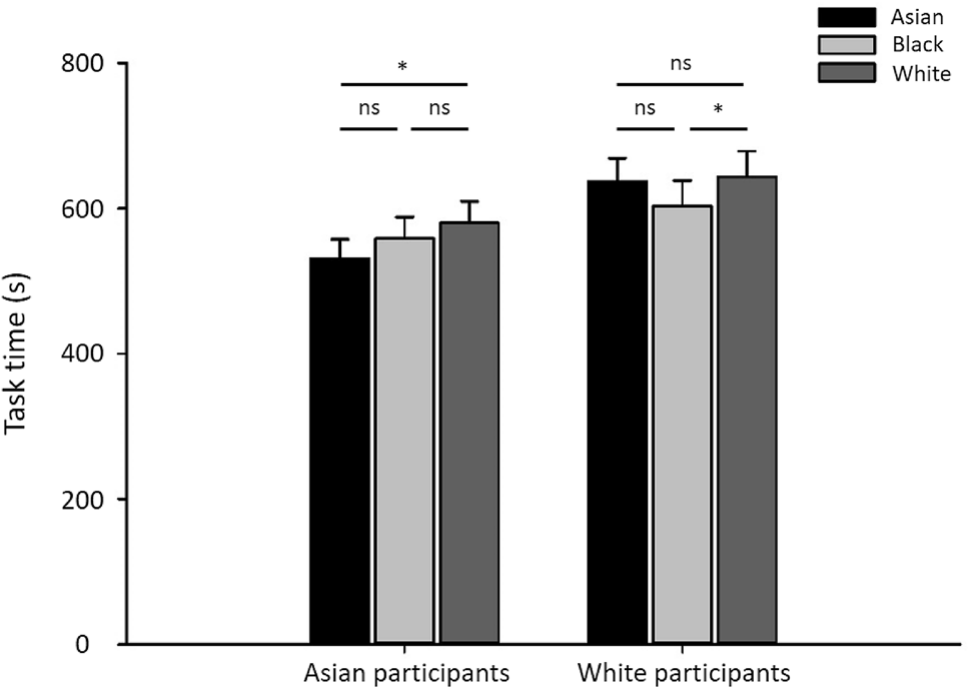
Task time for all matching tasks. There was no consistent evidence that participants spent more time on own-race faces compared to other-race faces. Error bars show + 1 SEM. *p <  0.05.

Finally, we determined whether the ORE could be explained by participants spending more time on own-race face tasks. Figure 4 shows the time spent for the face matching tasks. There was no significant interaction between Stimulus Race and Participant Race (F(2, 279) = 2.488, p = 0.085, Partial Eta Squared = 0.18). For Asian participants, task time on Asian face trials was significantly less than for White face trials (t(69) =  − 2.43, *p* < 0.05, d = 0.21), but there was no significant difference with Black face trials (t(69) =  − 1.59, p = 0.117, d = 0.118). There was no significant difference between task time for Black and White face trials (t(69) =  − 0.99, p = 0.324, d = 0.086). For White participants, the task time for White face trials was not significantly different compared to Asian face trials (t69) = 0.29, p = 0.772, d = 0.020), but was significantly higher for Black face trials (t(69) = 2.358, *p* < 0.05, d = 0.115). There was no significant difference between tasks times of Asian and Black (t(69) = 1.34, p = 0.174, d = 0.103) faces. There was also no difference task time for Black faces between Asian and White participants (t(69) =  − 0.936, p = 0.352, d = 0.135). Overall, there does not seem to be any consistent evidence that participants spent more time on own-race compared to other-race faces.

### Sorting task

**Figure 5:**
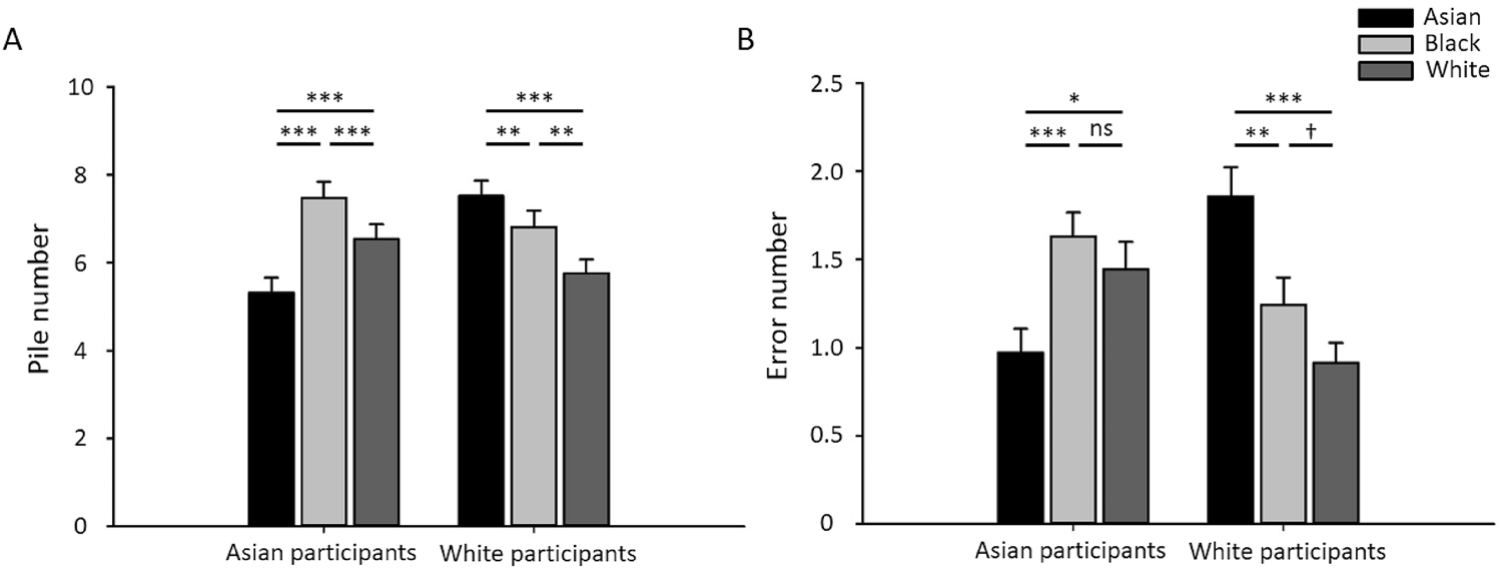
Performance on sorting task. Average pile number and Average wrong pile number made by Asian and White participant in Card sorting test. Error bars show + 1 SEM. ***p < 0.001, **p < 0.01, *p < 0.05, †, 0.01.

Figure 5 shows mean performance on the sorting task for Asian and White participants. There was a clear ORE for both participant groups. Asian participants generated fewer piles with Asian faces (mean ± SEM: 5.3 ± 0.3) compared to White (mean ± SEM: 6.5 ± 0.3; t(69) =  − 4.03, *p* < 0.001, d = − 0.48) and Black (mean ± SEM: 7.5 ± 0.4; t(69) =  − 6.33, *p* < 0.001, d = − 0.76) faces. Asian participants also made fewer errors with Asian faces (mean ± SEM: 1.0 ± 0.1) compared to White (mean ± SEM: 1.4 ± 0.2; t(69) =  − 2.532, *p* < 0.05, d = − 0.30) and Black (mean ± SEM: 1.6 ± 0.1; t(69) =  − 4.14, *p* < 0.001, d = − 0.50) faces. Similarly, White participants generated fewer piles with White faces (mean ± SEM: 5.8 ± 0.3) compared to Asian faces (mean ± SEM: 7.5 ± 0.3; t(69) =  − 5.24, *p* < 0.001, d = − 0.63) and Black faces (mean ± SEM: 6.8 ± 0.4; t(69) =  − 3.07, *p* < 0.01, d = − 0.37). White participants also made fewer errors with White faces (mean ± SEM: 0.9 ± 0.1) compared to Asian faces (mean ± SEM: 1.9 ± 0.2; t(69) =  − 5.94, *p* < 0.001, d = − 0.71) and Black faces (mean ± SEM: 1.2 ± 0.2; t(69) =  − 1.89, p = 0.062, d = − 0.23). We then asked if time in the UK could explain the size of the ORE in Asian participants. We found that there was no significant correlation in Asian participants between the time spent in the UK and the difference in pile number (r = − 0.014, p = 0.907) or errors (r = 0.093, p = 0.444) between Asian and White faces.

**Figure 6:**
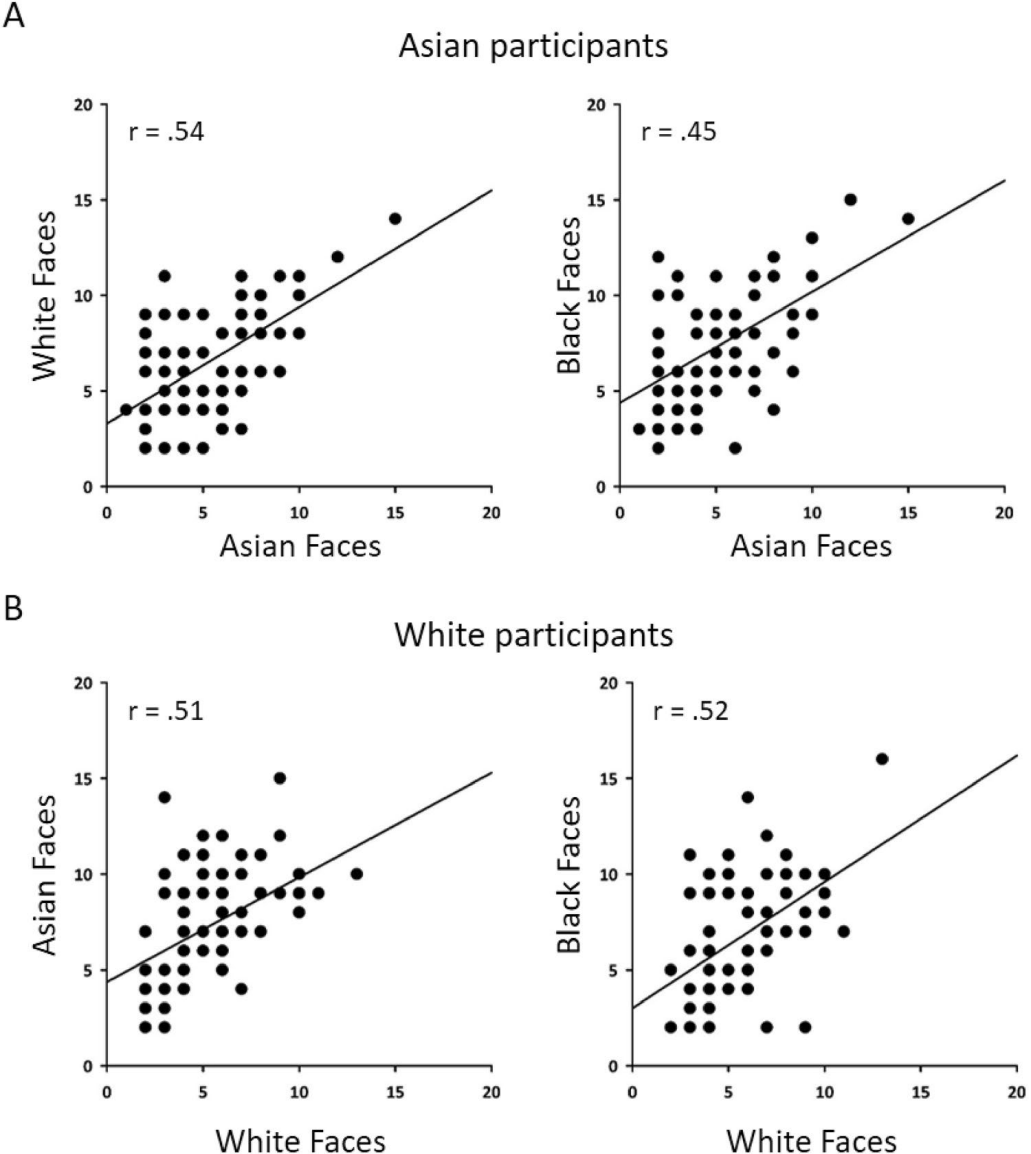
Correlation between pile number across different card sorting tasks in Asian and White participants. Significant positive correlations were found for each sorting task for both own-race and other-race faces suggesting that performance on own-race faces predicted performance on other-race faces.

Next, we compared individual differences on the different sorting tasks to determine whether performance with own-race faces predicted performance with other-race faces. We found performance on own-race faces was positively correlated with other-race faces (Fig. 6). For Asian participants, performance with Asian faces was positively correlated with White (pile: *r*_*s*_ = 0.54, *p* < 0.001; error: *r*_*s*_ = 0.18, p = 0.139) and Black (pile: *r*_*s*_ = 0.45, *p* < 0.001; error: *r*_*s*_ = 0.20, p = 0.106) faces. For White participants, performance with White faces was positively correlated with Asian (pile: *r*_*s*_ = 0.51, *p* < 0.001; error: *r*_*s*_ = 0.42, *p* < 0.001) and Black (pile: *r*_*s*_ = 0.52, *p* < 0.001; error: *r*_*s*_ = 0.18, p = 0.138) faces. This suggests that performance on own-race faces predicts better performance on other-race faces.

We then asked whether performance on ‘putting faces together’ (pile number) is correlated with performance on ‘telling faces apart’ (error number). If performance on these measures is related, we would expect a significant positive correlation. For Asian participants, there was a significant positive correlation between pile number and errors for Asian faces (*r*_*s*_ = 0.285, *p* < 0.05). However, there was significant negative correlation between these two measures for Black faces (*r*_*s*_ = − 0.256, *p* < 0.05) and no significant correlation for White faces (*r*_*s*_ = 0.021, p = 0.856). For White participants, there was no significant correlation between pile number and errors for White faces (*r*_*s*_ = − 0.152, p = 0.209) and Black faces (*r*_*s*_ = − 0.044, p = 0.717), but there was a significant negative correlation for Asian faces (*r*_*s*_ = − 0.300, *p* < 0.05). Overall, there did not seem to be any consistent relationship between the ability to put faces together and the ability to tell faces apart.

**Figure 7:**
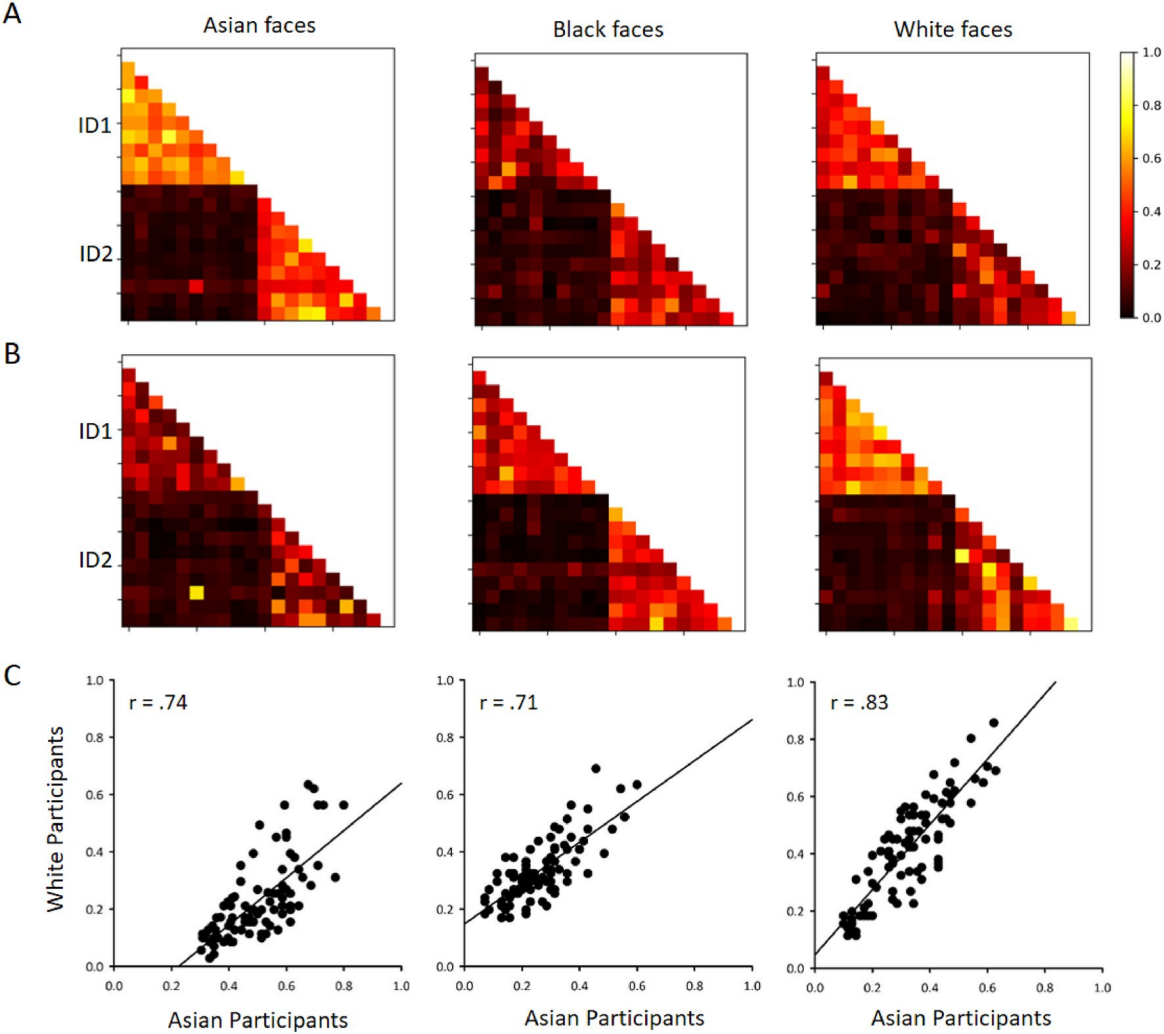
The probability of images being sorted into same pile on each sorting task for (**A**) Asian and (**B**) White participants. (**C**) Correlation of the probability of images being sorted into same pile for within identity images between Asian and White participants.

In our next analysis of the sorting tasks, we compared the way in which the participants sorted individual items on the own-race and other-race face tasks. Figure 7A,B shows the probability that each pair of images was sorted into the same pile. Participants typically sorted images into piles with the same identity, consistent with the low number of errors shown in Fig. 5. For Asian participants, the probability that two images with the same identity were placed in the same pile was significantly higher than the probability of two images from a different identity being placed in the same pile with Asian faces (within-person: 0.50 ± 0.013; between-person: 0.06 + 0.004; t(106.8) =  − 33.2, *p* < 0.001), Black faces (within-person: 0.26 ± 0.012; between-person: 0.06 ± 0.004; t(107.8) =  − 16.0, *p* < 0.001) and White faces (within-person: 0.32 ± 0.014; between-person: 0.07 ± 0.004; t(105.6) =  − 17.7, *p* < 0.001). Similarly, for White participants, the probability that two images with the same identity were placed in the same pile was significantly higher than for two images from a different identity with White faces (within-person: 0.41 ± 0.018; between-person: 0.07 ± 0.004; t(97.6) =  − 18.8, *p* < 0.001), Asian faces, (within-person: 0.22 ± 0.015; between-person: 0.07 ± 0.007; t(131.5) =  − 9.9, *p* < 0.001) with Black faces (within-person: 0.34 ± 0.011; between-person: 0.04 ± 0.004; t(110.4) =  − 24.6, *p* < 0.001).

To determine whether the pattern of sorting was consistent across the two participant groups, we measured the similarity of the sorting matrices between Asian and White participants (Fig. 7C). This was performed separately for within-identity and between-identity matches. The pattern of sorting between Asian and White participants was highly correlated for same-identity faces in all three tasks (Asian: *r*_*s*_ = 0.738, *p* < 0.0001; Black: *r*_*s*_ = 710, *p* < 0.0001; White : *r*_*s*_ = 0.826, *p* < 0.0001). Significant correlations were also evident for between-identity comparisons (Asian: *r*_*s*_ = 0.542, *p* < 0.0001; Black: *r*_*s*_ = 0.488, *p* < 0.0001; White: *r*_*s*_ = 294, *p* < 0.01). This shows that participants from both races sorted the faces in a similar way.

**Figure 8:**
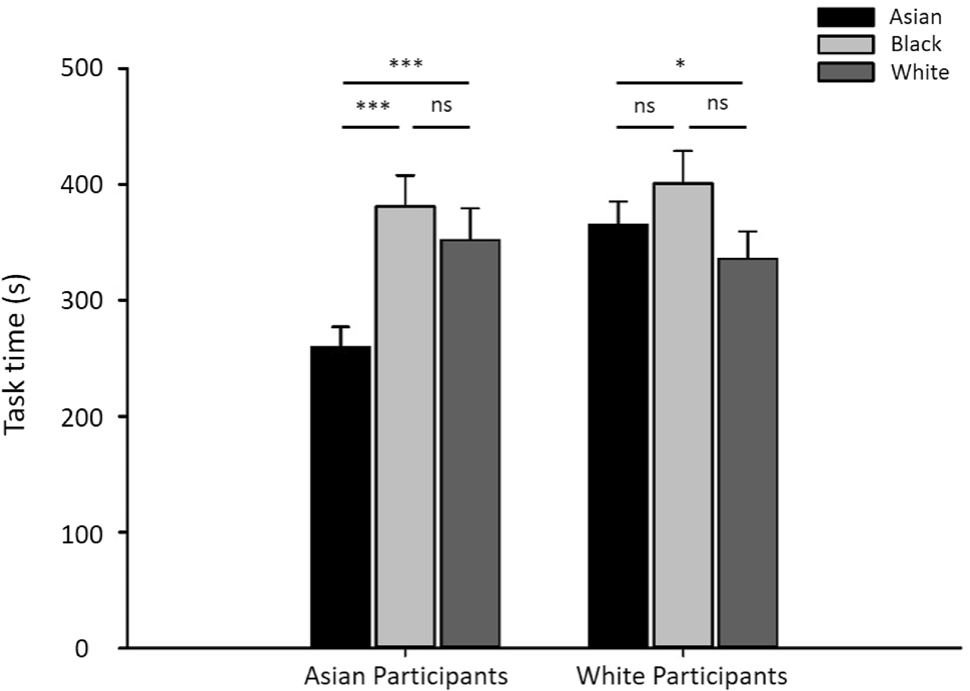
Task time of sorting tasks. There was no evidence that participants spent more time on own-race faces. Error bars show + 1 SEM. ***p < 0.001, **p < 0.01, *p < 0.05, †, 0.01.

Finally, we compared the time spent on each sorting task (Fig. 8). There was a significant interaction between stimulus race and participant race (F(2, 279) = 12.414, p = 0.001, Partial Eta Squared = 0.053). For Asian participants, task time with Asian faces was significantly lower than with both Black (t(69) =  − 5.17, *p* < 0.001, d = 0.642) and White (t(69) =  − 2.43, *p* < 0.001, d = 0.478) faces. There was no significant difference between time spent with Black and White faces (t(69) =  − 0.99, p = 0.324, d = 0.086). For White participants, task time with White faces was not significantly different to Asian faces (t(69) =  − 1.24, p = 0.219, d = 0.161), but was longer than with Black faces (t(69) =  − 3.65, *p* < 0.01, d = 0.300). There was no significant difference between task time with Asian and Black faces (t(69) =  − 1.54, p = 0.129, d = 0.176). There was also no difference task time for Black faces between Asian and White participants (t(69) = 0.421, p = 0.675, d = 0.075). Overall, there was no evidence that participants spent more time on own-race compared to other-race faces.

### Comparison of face matching and card sorting tasks

Next, we measured the covariation across behavioral measures in the matching and sorting tasks. Beginning with measures of ‘putting faces together’, we compared same-identity performance on the matching task with numbers of piles on the sorting task. Our prediction was that this should be negatively correlated if these measures are related. In other words, higher accuracy on judging whether two face images from the same identity are the same person in the matching task should be linked to a greater ability to group faces in the sorting task. For Asian participants, there was a significant negative correlation for Asian faces (*r*_*s*_ = − 0.520, *p* < 0.001). There was a significant negative correlation for Black faces (*r*_*s*_ = − 0.272, *p* < 0.05), but the correlation was not significant for White faces (*r*_*s*_ = − 0.158, p = 0.191). For White participants, there was a significant negative correlation for White faces (*r*_*s*_ = − 0.289, *p* < 0.05), but also for Asian (*r*_*s*_ = − 0.442, *p* < 0.001) and Black (*r*_*s*_ = − 0.339, *p* < 0.01) faces. Overall, this provides evidence that the ability to put faces together covaries across these two tasks.

To determine covariation in the ability to ‘tell faces apart’, we compared performance on the different-identity trials of the matching tasks with numbers of errors on the sorting task. Again, if these measures were related, a negative correlation is predicted. In other words, if participants are more accurate in determining that two faces from different identities are different in the matching task, they should make fewer errors in the sorting task. For Asian participants, there was a significant negative correlation for Asian faces (*r*_*s*_ = − 0.296, *p* < 0.05), but also for Black (*r*_*s*_ = − 0.313, *p* < 0.01) and White (*r*_*s*_ = − 0.251, *p* < 0.05) faces. For White participants, there was a significant negative correlation for White faces (*r*_*s*_ = − 0.257, *p* < 0.05). There was also a significant negative correlation for Asian (*r*_*s*_ = − 0.286, *p* < 0.05), but not for Black (*r*_*s*_ = − 0.019, p = 0.877) faces. Overall, this shows evidence for covariance in the ability to tell faces apart across the two tasks.

Finally, we asked whether performance on the different measures of putting faces together or telling them apart could be predicted by the time participants spent on each task. On the matching task, there were no significant correlations between time and different identity trials with Asian participants (Asian: r = − 0.013, p = 0.913; Black: r = 0.187, p = 0.120, White: r = 0.186, p = 0.124) or White participants (Asian: r = 0.031, p = 0.796; Black: r = 0.010, p = 0.932, White: r = − 0.030, p = 0.808). There was no consistent relationship between time and accuracy on same identity trials for Asian participants (Asian: r = 0.056, p = 0.644; Black: r = 0.275, p = 0.021, White: r = 0.161, p = 0.183) or White participants (Asian: r = 0.492, *p* < 0.001; Black: r = 0.239, p = 0.046, White: r = 0.332, p = 0.005). On the sorting task, there were no significant correlations between time and errors with Asian participants (Asian: r = 0.095, p = 0.433; Black: r = − 0.037, p = 0.762, White: r = − 0.009, p = 0.942) or White participants (Asian: r = − 0.154, p = 0.204; Black: r = − 0.138, p = 0.254, White: r = − 0.016, p = 0.898). There was also no consistent relationship between time and the number of piles for Asian participants (Asian: r = 0.292, p = 0.014; Black: r = − 0.083, p = 0.495, White: r = − 0.081, p = 0.503) or White participants (Asian: r = − 0.245, p = 0.041; Black: r = 0.128, p = 0.292, White: r = 0.172, p = 0.153).

## Discussion

Our results provide clear evidence for the ORE on two tasks of face recognition: matching and sorting. We found that Asian participants performed better on Asian compared to White faces, whereas White participants performed better on White compared to Asian faces. Despite clear evidence for an ORE, we found that overall performance on own-race faces significantly predicted overall performance on other-race faces in both the matching and sorting tasks. That is, more accurate performance on own-race faces predicted more accurate performance on other-race faces. The strong covariation in performance across individuals from different races on own-race and other-race faces suggests that similar perceptual processes are used in the perception of own-race and other-race faces.

A dominant theory of the ORE proposes that other-race faces are processed in qualitatively different ways^13–15^. Own-race faces due to their in-group status are processed at an individual level, whereas other-race faces due to their out-group status are processed at a more categorical level^16,17^. Thus, the perception of own-race and other-race faces is different depending on the outcome of the preceding racial categorization. Our results showing the covariation in overall performance with own-race and other-race faces suggests that similar perceptual processes are used for all face tasks, regardless of race. Previous studies have also found covariation in overall performance on own-race and other-race faces^7,8,33–36^. This covariation in performance is consistent with studies involving participants with below average (developmental prosopagnosics) or above average (super-recognizers) face recognition based on own-race faces, with both groups showing an ORE^35,36^. Other studies have shown that individual differences in holistic processing can predict the size of the ORE^33,37^. Together these findings are consistent with the idea that own-race and other-race faces are processed using the same perceptual mechanisms.

Although these findings argue against the idea that qualitatively different processes (e.g. categorization vs individuation) explain the ORE, it is possible that the covariation in the overall performance across individuals shown here and in previous studies reflects a difference in motivation or general perceptual ability. To address this issue, we measured covariation across different dependent variables within each task. For example, in the matching task, we measured performance on same and different trials separately. We found that performance on either same trials or different trials with own-race faces predicted the corresponding measure with other-race faces. Interestingly, we found no consistent covariation between individual performance on same identity trials and different identity trials. This suggests that the processes that lead to these judgements are to some extent independent and would appear to rule out the possibility of an explanation based on motivation or general perceptual ability.

The difference in recognition of own-race and other-race faces suggests that we are differentially sensitive to differences in the variation of faces from different races^38,39^. This could mean that the way in which participants perceive faces from different races is qualitatively different. To address this issue, we used an item analysis to compare the patterns of response. We found a similar covariation in performance between participants from different races in an item analysis. For example, trials on a matching task (irrespective of face race) that were found to be difficult for Asian participants were also found to be difficult for White participants, whereas trials that were easier for Asian participants were also easier for White participants. For the sorting task, we found that the pattern of sorting was very similar for participants from different races, irrespective of the face race. That is, faces that were more often put in the same pile by White participants were also more likely to be put in the same pile by Asian participants. The similarity in the pattern of response again suggests that similar mechanisms are used for the perception of own-race and other-race faces. This fits with a recent study showing that shape and texture information from faces is used in a similar way for the recognition of own-race and other-race faces^40^.

All the tasks in this study were self-paced. This allowed us to ask whether participants spent more time on own-race faces. A prediction from social group theories of the ORE suggests that other-race faces are processed with lower levels of attention and motivation compared to own-race races. However, we found no consistent evidence for participants spending more time on own-races faces. These findings fit with previous studies which have measured self-reported effort^34^ or time-spent^41^ on own-race and other-race faces and fail to find any bias toward own-race faces. In fact, these results agree with our findings that, if anything, participants spend more time on other-race face tasks. Presumably this reflects the fact that these tasks are more challenging. Taken together, the covariation in performance on tasks involving own-race and other-race and lack of any bias in task time for own-race faces suggests that the ORE cannot be accounted for by a difference types perceptual processing that result from social categorization.

An alternative theory of the ORE is that it is based on differential exposure to same-race and other-race faces^23–26,31^. Support for the role of experience comes from developmental studies showing that the ORE increases with age, presumably as a function of increased experience^11,22,31,42^. This leads to recognition being optimized for processing variance in own-race faces. Nevertheless, the same perceptual mechanisms are used to perceive own-race and other-race faces, it is just more tuned to own-race faces. This suggests that a similar type of processing is used to perceive faces regardless of race. A strong prediction is that individual performance on own-race and other-race faces should covary. Our results provide support for this prediction. Another prediction from this theory is that the ORE should vary as a function of exposure to other-race faces. We found a negative correlation between the duration that East Asian participants were in the UK and the difference in performance on own-race and other-race faces in the matching task, but not in the sorting task. Although this provides support for the role of perceptual experience, this does not rule out the possibility of some role for group-bias in natural viewing. For example, a reduced motivation to interact with individuals from an out-group (such as people from a different race) could result in reduced perceptual experience^24^. This would then cause differences in experience that give rise to the perceptual differences reported here and previous studies of recognition.

In this study, we had tasks that involved Asian and White faces using Asian and White participants. This part of the design is critical in studies of the ORE in order to show a cross-over interaction. This is important to rule out the possibility the potential confound of differences in task difficulty. The potential problem is evident in Fig. 3 which shows Asian participants have higher performance for Asian faces and White participants have higher performance for White faces. Nonetheless, if only Asian participants had been tested then performance would appear to be similar across all tasks. This might have led to the wrong conclusion that there is not an ORE. However, the actual reason is that the Asian face task is harder than the White face task. We also addressed this issue by including in our design Black faces that were other-race for both Asian and White participants. Across the two tasks, performance on Black faces was higher for White compared to Asian participants. One possible explanation for this finding is the higher proportion of the population who are Black in the UK compared to in China^43,44^. This could also be related to different levels of group bias as a result of limited interactions. However, if this were the case then we would expect that Asian participants should spend less time on the Black face tasks compared to White participants and there is no evidence for any difference in task time. Rather, it would seem that the difference between Asian and White participants with Black faces may reflect differences in perceptual experience.

Tasks measuring ability in face recognition require participants to determine whether faces are from the same person (putting faces together) and whether they are from different people (telling faces apart). First, we asked whether measures of the ability to ‘put faces together’ across the two tasks were correlated. Because lower numbers of piles on the sorting task and higher accuracy on the matching task reveal higher performance, we predicted significant negative correlations if the ability to ‘put faces together’ was correlated across the two tasks. Consistent with this prediction, we found significant negative correlations across in all but one of the different combinations of participant and face race. Next, we asked whether measures of ‘telling faces apart’ in the two tasks were correlated. Again, because low numbers of errors on the sorting task, but high levels of accuracy on the matching task indicate higher performance, we predicted significant negative correlations if the ability to ‘tell faces apart’ covaried across the two tasks. We found that performance on different-identity trials in the matching task was negatively correlated with number of piles in all but one of the different combinations of participant and face race. These results suggest that corresponding sub-processes are involved in both the matching and sorting tasks.

Finally, The ORE has often been framed as a problem with individuating (discriminating between) other-race faces, consistent with the claim that other-race faces all look similar^45,46^. However, we found that there was no difference in the proportion of responses (irrespective of accuracy) in the matching task. Moreover, in the sorting task, participants made more piles rather than less (see also^9^. This suggests that rather than all looking the same, other-race faces look more different.

In conclusion, we found that participants were more accurate with own-race faces compared to other-race faces in a matching task. Despite a clear ORE, performance on own-race faces was positively correlated with performance on other-race faces. The ORE could not be explained by different levels of attention or motivation, as participants did not spend more time with own-race faces compared to other-race faces and that different measures from each task covaried independently. Together, these findings suggest that own-race and other-race faces engage the same perceptual mechanisms and argues against the theory that group bias causes own-race and other-race faces to engage different cognitive mechanisms.

## Methods

### Participants

We recruited an opportunity sample of 140 participants who were students from the University of York (70 Asian: 59 female, mean age: 24.2 and 70 White: 58 female, mean age: 20.3) for this study. The participant numbers significantly exceed those used in related studies of the ORE to provide sufficient power for the analyses. Critically, we used a cross-over design that required recruiting double the number of participants. All Asian and White participants had grown up in East Asian and Western European countries, respectively. For Asian participants, their average time in the UK period was about 13 months (Mean ± SEM: 12.9 ± 2.08). All participants gave their written informed consent. White participants did not have any experience of living in an Asian country. The study was approved by the Psychology Ethics Committee at the University of York and was performed in accordance with the Declaration of Helsinki. All participants took part in Experiment 1 and 2. Participants were given course credit for taking part in the experiments.

### Matching tasks

There were three face matching tasks that were composed of images from either Asian, Black or White male faces. Each matching task had 90 trials. In each trial, a pair of face images was presented together (Fig. 1). In half of the trials, the faces were from the same identity and in the remaining half of the trials the faces were from a different identity. The order of tasks was randomized and counterbalanced across all participants. There was no time restriction for each task, but the time spent on each task was recorded.

Images for the White matching task were taken from an existing test^47^. The images for the Asian and Black matching tasks were taken from a variety of sources on the internet. The criteria for image selection was that they showed the face in roughly frontal aspect, were free from occlusions and did not show any clear facial expression. Other than these restrictions, the images were free to vary in a way that reflects the variability found in natural viewing. The images were cropped to 158 × 222 pixels. Participants viewed images at a distance of approximately 57 cm, such that each image subtended 7.8 × 10.2 degrees of visual angle.

Participants performed this task in person. They were asked to indicate whether each pair of faces was from the same identity or a different identity. Participants wrote their answers on a sheet. The task was self-paced, but the time spent on each task was recorded. We measured discriminability (d’)^48^, by calculating hits (trial: same identity, response: same), misses (trial: same identity, response: different), false positives (trial: different identity, response: same) and correct rejections (trial: different identity, response: different). To further explore the pattern of performance for the two race groups in matching tasks, performance on same-identity and different-identity faces was determined separately for each task and participant group. No participants were removed from the analysis as a result of poor data quality.

### Sorting tasks

There were three sorting tasks with images of either Asian, Black or White male faces. Each task had 20 images with 10 images from one identity and 10 images from a different identity. Images from the sorting task were different from those you in the matching task. However, we used the same criteria for image selection as for the matching tasks. Images were cropped to a size of 158 × 222 pixels, printed in gray scale to a size of 7.3 × 5.6 cm and then laminated (Fig. 2). For each sorting task, participants were given a shuffled stack of the 20 face images. They were instructed to sort the faces into piles that had the same identity. Participants performed this task in person. The dependent measures were the number of piles and the number of errors (more than one identity in a pile). There was no time restriction for each task, but the time spent on each task was recorded. No participants were removed from the analysis as a result of data quality.
